# Revision lateralization osteotomy of the tibial tubercle has excellent outcomes in patients suffering from previous medial tibial tubercle overcorrection

**DOI:** 10.1007/s00402-022-04700-1

**Published:** 2022-11-23

**Authors:** Sebastian Gebhardt, Alexander Zimmerer, Felix Zimmermann, Georgi I. Wassilew, Peter Balcarek

**Affiliations:** 1 Center for Orthopaedics, Trauma Surgery and Rehabilitation Medicine, University Medicine Greifswald, Ferdinand-Sauerbruch-Straße, 17475 Hansestadt Greifswald, Germany; 2 ARCUS Sportsclinic, Rastatterstr. 17-19, 75179 Pforzheim, Germany; 3 BGU Hospital, Ludwig-Guttmann-Straße 13, 67071 Ludwigshafen, Germany

**Keywords:** Tibial tuberosity osteotomy, Patellofemoral pain, Patellofemoral osteoarthritis, Medial patella maltracking, Patella medialization

## Abstract

**Introduction:**

Tibial tubercle osteotomy (TTO) is a common procedure used to treat patients with patellofemoral instability (PFI) and osteoarthritis (PFOA). Medial patellar maltracking due to previous excessive medialization of the tibial tubercle has rarely been reported. Therefore, the goal of this study was to assess patient-reported outcome measures (PROMs) after revision osteotomy with lateralization of the tibial tubercle (RL-TTO) to correct medial patellofemoral maltracking.

**Materials and methods:**

Between 2017 and 2021, a series of 11 patients (male/female 1/10; age 35.8 ± 10.5 years) were treated by RL-TTO, of whom 8 patients could be retrospectively evaluated after a mean of 32.4 ± 15.1 months (range 18–61 months) postoperatively. The Kujala anterior knee pain scale, the patellofemoral subscale of the Knee Osteoarthritis and Outcome Score (KOOS-PF), and a numeric analog scale (NAS; 0–10) regarding anterior knee pain (AKP) at rest and during activity were assessed from pre- to postoperatively.

**Results:**

The preoperative mean tibial tubercle-trochlear groove (TT-TG) and tibial tubercle-posterior cruciate ligament (TT-PCL) distances were − 6.5 ± 6.5 mm and 0.7 ± 4.6 mm, respectively. The intraoperatively determined amount of tibial tubercle lateralization averaged 10.7 ± 3.6 mm. The Kujala score and KOOS-PF improved significantly from 33.6 ± 10.1 (23–51) points to 94.4 ± 6.2 points (82–100) (*p* < 0.001) and from 20.6 ± 13.2 points (0–43.3) to 87.3 ± 9.9 points (72.8–100) (*p *< 0.001) from pre- to postoperatively, respectively. Pain at rest decreased from 5.8 ± 1.9 to 0.8 ± 0.9 (*p* < 0.001), and pain during activity decreased from 8.6 ± 1.3 to 1.6 ± 1.5 (*p* < 0.001).

**Conclusion:**

RL-TTO significantly improved subjective knee function and AKP in patients suffering from medial patellar maltracking due to previous excessive tibial tubercle medialization osteotomy at short-term follow-up.

## Introduction

Various tibial tubercle osteotomy (TTO) techniques for distalization, medialization or anteromedialization have been described [1]. Indications for TTO include anterior knee pain (AKP), patellofemoral osteoarthritis (PFOA) or patellofemoral instability (PFI) as a result of patella alta or lateral patellar maltracking. Predominantly good to excellent results were reported in short- and midterm follow-up [2–4]. However, long-term follow-up studies documented a deterioration of functional outcome scores and signs of PFOA progression [5–7].

The integrity of the medial patella facet and the corresponding trochlear joint surface is thought to play an important role in achieving satisfactory results after TTO [8]. Besides recurrent instability, wound infection, thromboembolic events and nonunion of the osteotomy were identified as the main sources of complications after TTO surgery [9–11]. In addition, signs of early- and late-stage osteoarthritis were observed in 54 and 8% of patients after 12 years, respectively [6, 7].

**Fig. 1:**
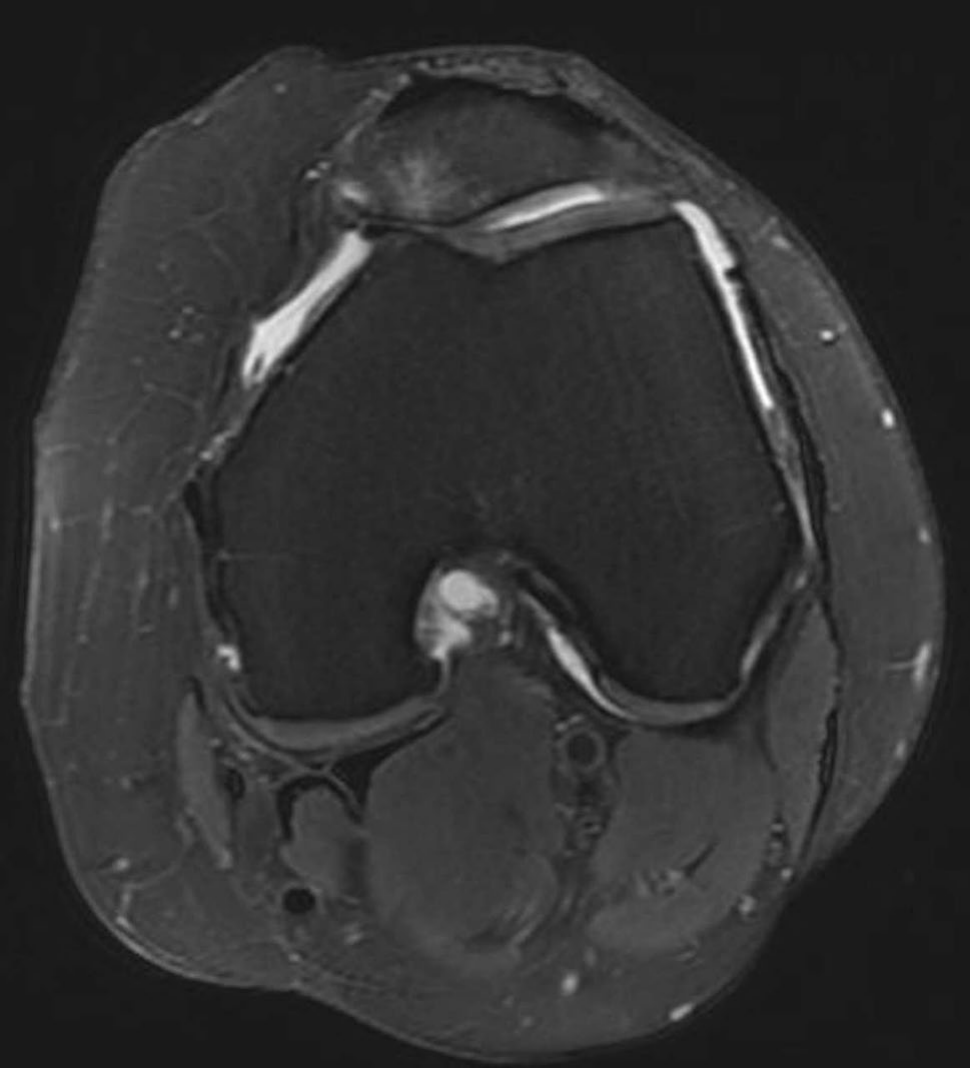
Transverse MRI slice showing medialized positioning of the patella with consecutive medial patellofemoral cartilage damage and bone bruise at the medial patellar facet

Medial overcorrection of the tibial tubercle might lead to abnormal patellar tracking with increasing medial patellofemoral joint contact pressure. However, indications, procedures and outcomes of revision surgery have not been reported to date. A certain number of patients with AKP, reduced knee joint function, and medial patellofemoral chondral lesions presented to our clinic at a mean of 10 years after primary TTO. Investigations showed a massive medialization of the tibial tubercle with pathological low tibial tuberosity—trochlear groove (TT-TG) and tibial tuberosity—posterior cruciate ligament (TT-PCL) distances, resulting in medialized patella tracking (Fig. 1), a complication that has only occasionally been reported. [12] Therefore, the aim of this study was to assess the results of revision osteotomy with lateralization of the previously medialized tibial tubercle (RL-TTO) to improve medial patellar maltracking. The hypothesis was that RL-TTO would lead to improvement in patient-reported outcome measures (PROMs) and reduction of AKP.

## Materials and methods

This retrospective investigation included 8 out of 11 patients who were treated by RL-TTO between January 2017 and October 2021. Patient demographics and surgical treatments are shown in Table 1. Revision surgery was scheduled in patients with a history of operative medialization of the tibial tubercle, AKP and radiographic measures documenting a TT-TG distance of ≤ 0 mm or a TT-PCL distance of ≤ 5 mm on standard MRI sequences of the knee. The medial position of the patella was accompanied by signs of chondromalacia at the medial patella facet or at the corresponding trochlea surface. The senior author performed all clinical and radiological evaluations as well as the surgical treatments. Approval was obtained from the local ethics committee (F-2019-070).

**Table 1:** Demographic data, details on the performed operation and patellofemoral cartilage condition as intraoperatively documented

Patient	Sex	Age	Index OP to revision (months)	Operation performed	FU after RL-TTO (months)	Cartilage condition (Outerbridge)	
						Medial Trochlea	Medial Patella
1	F	28	80	RL-TTO, D-TTO, MPFL-R, CD-P	–	4	3
2	F	43	59	RL-TTO, D-TTO, MD-T	34	4	3
3	F	55	117	RL-TTO, P-TTO, CD-P	35	3	2
4	F	18	37	RL-TTO, CBT	30	0	0
5	F	25	82	RL-TTO, DFO, CD-P	44	0	2
6	F	28	90	RL-TTO, CBT, MPFL-R	61	0	0
7	M	31	43	RL-TTO, MCI-P, Z-plasty	18	0	3
8	F	27	108	RL-TTO, MD-T, SP	18	4	4
9	F	43	247	RL-TTO, CBT, MD-FC, CD-P	18	4	3
10	F	37	226	RL-TTO, D-TTO, MPFL-R, MD-T	–	3	2
11	F	43	255	RL-TTO, MPFL-R, MD-T, CD-FC	–	4	4
Mean ± SD		35.8 ± 10.5	122.1 ± 81.3		32.4 ± 15.1	2.4 ± 1.9	2.4 ± 1.4

*SD* standard deviation, *F* female, *M* male, *RL-TTO* revision lateralization tibial tubercle osteotomy, *D-TTO* distalization tibial tubercle osteotomy, *MPFL-R* medial patellofemoral ligament reconstruction, *CD-P* cartilage debridement at the patella, *MD-T* micro drilling at the trochlea, *P-TTO* proximalization-tibial tubercle osteotomy, *CBT* cancellous bone transfer, *DFO* derotational femoral osteotomy, *MCI-P* minced cartilage transplantation at the patella, *MD-FC* micro drilling femoral condyle, *CD-FC* cartilage debridement at the femoral condyle

### Surgical procedure

**Fig. 2:**
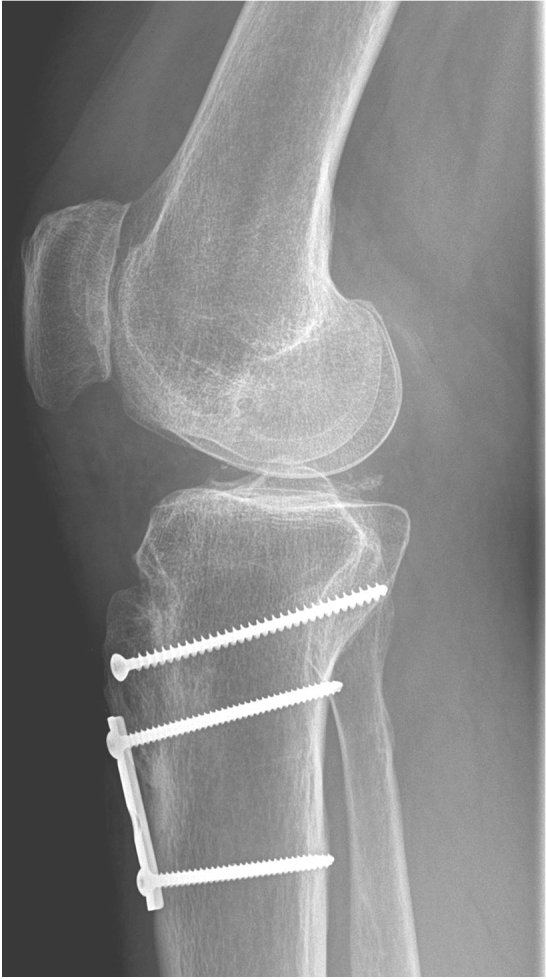
Example lateral X-Ray of a standard fixation of the tibial tuberosity fragment using a one-third-tubular plate, proximal lag screw and two distal cortical screws

Patients were placed in the supine position. An electric leg holder was used to allow free range of motion during surgery. Initial diagnostic arthroscopy using standard portals was performed to control for intraarticular lesions, to document patella tracking, to address possible soft tissue adhesions, and to treat cartilage lesions. Thereafter, the skin was incised alongside the formerly used surgical approaches on the anterior tibial head at a length of approximately 8 cm. After thorough preparation of subcutaneous tissue and fascia, the noticeably medially placed tibial tubercle and distal part of the patella tendon were displayed. The osteotomy was carried out by an oscillating saw and chisels creating a fragment measuring approximately 2–3 cm in width, 6–7 cm in length and 1 cm in thickness, as recommended elsewhere [2, 13, 14]. The tibial tubercle was detached from its bed and mobilized by carefully incising the medial and lateral retinacula until the osteotomy fragment could be moved freely. The tibial tubercle fragment was lateralized 10–15 mm depending on the preoperative evaluation of TT-TG and TT-PCL distances. Depending on the bony integrity, transfer of cancellous bone from the proximal tibia head was performed as needed. Debridement of the former attachment area of the tibial tubercle was carried out, and two K-wires were used for temporary fixation of the tibial tubercle. The knee was then flexed at approximately 60 degrees, verifying central patellar tracking in the trochlea groove. In addition, patellar height was controlled under lateral fluoroscopy by checking for a Canton-Dechamps Index of approximately 1.0 also in 60 degrees of knee flexion. If necessary, the temporary position of the tibial tubercle was reoriented. For final fixation, the proximal K-Wire was removed and replaced by one small fragment lag-screw; thereafter, the distal *K* Wire was removed, and a one-third-tubular bridging plate with two cortical screws was used for secure refixation (Fig. 2). The retinacula were closed using resorbable suture material (2.0 Vicryl, Ethicon). Soft tissue balancing by z-plasty-lengthening of the retinacular structures was performed as needed.

### Postoperative treatment

Postoperatively, patients were immediately allowed full range of motion as tolerated. Weight bearing was limited to 15 kg for four weeks and gradually increased afterwards. Full weightbearing was allowed following clinical and radiographic control for union of the osteotomy approximately 6 weeks postoperatively. Isometric exercises under physiotherapeutic supervision and continuous passive motion (CPM) were started from day one postsurgery. Starting from 4 weeks postsurgery, closed chain exercises and focused quadriceps strengthening were allowed to prepare for return to sport after 6 months, if tolerated.

### Outcome measures

Patient-reported outcomes were evaluated using the Kujala anterior knee pain scale [15], the KOOS patellofemoral subscale (KOOS-PF) [16] and the numeric analog scale (NAS, 0–10 points) to assess the severity of AKP during rest and activity.

### Statistical analysis

The data were assessed for normality and are presented as the mean ± standard deviation (SD) and range. Paired two-tailed *t* tests were used to assess differences between the pre- and postoperative data. All analyses were performed using GraphPad Prism (version 9.3.1; GraphPad Software, San Diego, CA, USA). The level of significance was set to 0.05.

## Results

Between January 2017 and October 2021, 11 patients (M/F 1/10, mean age 35,8 ± 10,5 years) were diagnosed with medial overcorrection of the tibial tubercle and underwent RL-TTO. Three patients were lost to follow-up; the remaining 8 patients had a mean follow-up period of 32.4 ± 15.1 months (range 18–61 months). The mean time period between initial medialization osteotomy and revision surgery of all 11 patients was 122.1 ± 81.3 months. The documented cartilage damage (according to Outerbridge) at the time of revision surgery was graded 2.4 ± 1.9 for the medial trochlea groove and 2.4 ± 1.4 for the medial patellar facet. Individual data of each patient are presented in Table 1.

**Fig. 3:**
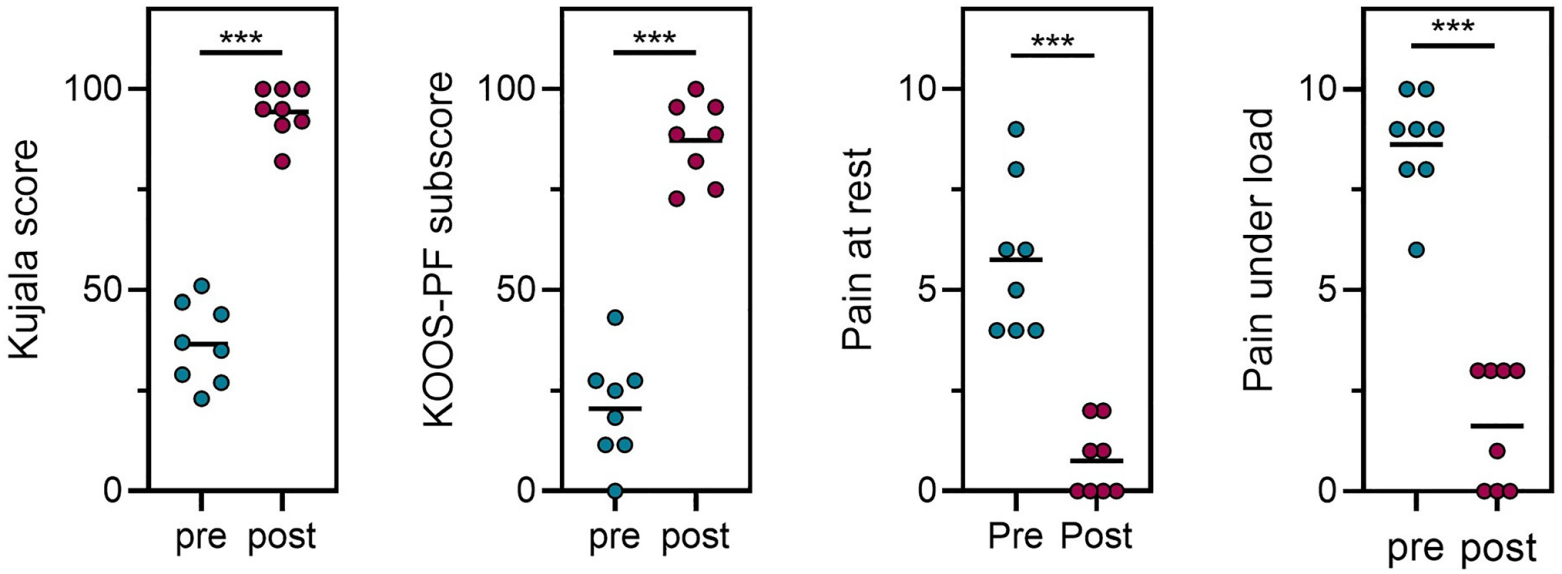
Pre- and postoperative comparison of Kujala score, KOOS-PF subscale, pain at rest and pain during activity (***p > 0.001, Graph edited with GraphPad Prism Version 9.3.1)

Prior to revision surgery the mean TT-TG and TT-PCL distances averaged − 6.5 ± 6.5 and 0.7 ± 4.6 mm, respectively. The average intraoperative amount of lateralization was 10.7 ± 3.6 mm. The Kujala score and KOOS-PF subscale score improved significantly from 36.6 ± 10.1 points (23–51) to 94.4 ± 6.2 points (82–100) and from 20.6 ± 13.2 points (0–43.3) to 87.3 ± 9.9 points (72.8–100) from pre- to postoperatively, respectively (both *p* < 0.001). Pain at rest decreased from 5.8 ± 1.9 to 0.8 ± 0.9 (*p *< 0.001), and pain during activity decreased from 8.6 ± 1.3 to 1.6 ± 1.5 (*p* < 0.001) (Table 2 and Fig. 3). Following surgery, one patient underwent a course of oral corticosteroid treatment for ongoing knee joint synovitis. Removal of hardware due to ongoing mechanical irritation over the tibial tubercle was performed in 6 patients 10–12 months postsurgery. No infection, delayed union or nonunion was observed. No cases of recurrent patellar instability occurred.

**Table 2:** Radiographic measures, intraoperative measures and patient-reported outcome measures before and after RL-TTO

Patient	Preop TT-TG Distance (mm)	Preop TT-PCL Distance (mm)	Intraop lateralization (mm)	Kujala Score		KOOS-PF		NAS pain at rest		NAS pain during activity	
				Preop	Postop	Preop	Postop	Preop	Postop	Preop	Postop
1	− 10	2,5	8	–	–	–	–	–	–	–	–
2	− 12	0	12	35	95	11.5	75	6	1	9	1
3	− 12	− 4	12	29	100	0	100	9	0	10	0
4	− 17	0	15	51	95	43.3	88.8	4	1	8	3
5	0	9	10	27	100	11.5	95.5	5	0	9	0
6	− 9	0	15	23	100	18.3	95.5	8	0	10	0
7	0	0	5	44	91	27.5	82	6	2	9	3
8	− 7	− 3	12	47	92	27.5	88.8	4	0	6	1
9	− 7	− 1	10	37	82	25	72.8	4	2	8	3
10	5	9	5	–	–	–	–	–	–	–	–
11	− 3	− 5	14	–	–	–	–	–	–	–	–
Mean	− 6.5 ± 6.5	0.7 ± 4.6	10.7 ± 3.6	36.6 ± 10.1	94.4 ± 6.2	20.6 ± 13.2	87.3 ± 9.9	5.8 ± 1.9	0.8 ± 0.9	8.6 ± 1.3	1.6 ± 1.5

*NAS* numeric analog scale, patients 1, 10 and 11 were lost to follow-up

## Discussion

The main finding of the study was that RL-TTO significantly improved subjective knee function and AKP in patients suffering from medial patellar maltracking due to previous excessive tibial tubercle medialization osteotomy.

Our findings and a number of earlier investigations [8, 17] suggest that inadequately restored biomechanics and, in particular, medial tibial tubercle overcorrection play a role in cartilage degeneration and deterioration of functional outcomes following TTO over time.

The iatrogenic medial malpositioning of the tibial tubercle reported in this case series has only rarely been discussed in the available literature on complications and outcomes following TTO [1, 9–12, 18, 19]. Thus, to our knowledge, this is the first investigation of patients suffering from medial tibial tuberosity overcorrection and outcomes following RL-TTO.

While lateral patellar maltracking and its risk factors including genu valgum, lateral position of the tibial tubercle, trochlear dysplasia, and increased femoral antetorsion have been widely discussed in the literature [20], medial patellar tracking is a rarely observed condition that to date has mainly been described as a complication of extensive lateral retinaculum release. [21] In contrast, all cases included in this study had an iatrogenic aetiology of medial patellar maltracking following an excessive TTO medialization procedure.

Several techniques for TTO exist, including the early medialization techniques described by Hauser et al. [22], the Roux-Elmslie–Trillat medialization technique [23], the anteromedialization technique described by Fulkerson [24], the self-centering medialization TTO [25], and procedures in which the tibial tubercle is completely detached to allow for concomitant medialization and distalization [26, 27].

Recurrent patellar dislocation following TTO has been reported in 5–12% of cases [11]. Payne et al. identified malunion (0.8%) and tibial fracture (1%) as major complications after TTO. The authors further distinguished between different TTO techniques and showed higher rates of nonunion (2.4%) and tibial fracture (2.5%) for techniques that completely detached the tibial tubercle [9]. Although many studies reported good results during short-term follow-up, some results tended to deteriorate over time. In this context, the technique described by Hauser et al. [22] has been reported with up to 70% of PFOA after 16 years postoperatively [5]. Remarkably, a high incidence of PFOA was found in young patients, suggesting that this kind of operation enhanced the development of PFOA [28]. Findings were linked to a degree of posterior displacement of the tibial tubercle that came with the medialization during Hauser’s procedure. During the Elmslie-Trillat procedure, the tibial tubercle is not displaced posteriorly [23]. Good and excellent results were observed in 71% of patients 4 years after surgery, which decreased to 62.5% at the 12 year follow-up, as signs of early- and late-stage osteoarthritis were observed in 54 and 8% of patients, respectively. [6, 7]

In an attempt to prevent increased pressure on the medial patellar facet, Fulkerson introduced the anteromedialization technique [24]. Although intended to reduce joint contact forces, this technique shifts the area of peak contact medially and proximally [29]. Accordingly, good to excellent results were observed in 87% of patients with distal or lateral patellar lesions, compared to 55% with a medial facet lesion, and 20% with proximal or diffuse lesions at a mean follow-up of 4 years [8]. In addition, a long-term follow-up study reported approximately 94% of subjective patient satisfaction 15 years after anteromedialization TTO in subjects with lateral patellofemoral chondromalacia [17].

The cartilage status at the medial patellar facet and the corresponding trochlea seem to influence long-term outcome after TTO. Patients included in this case series had a medial malpositioning of the tibial tubercle with an average TT-TG distance of − 6.5 mm resulting in low Kujala- and KOOS-PF scores at a mean of 10 years after initial surgery. In particular, this outcome is problematic considering the young age (35.8 years) of our study population. There was no information available on the exact TTO technique that was used for primary surgery as well as no data were given on TT-TG and TT-PCL distances prior to first TTO, which can be considered as crucial for determining the amount of required intraoperative tibial tubercle medialization [30, 31]. However, a high TT-TG distance alone does not automatically mean that TTO is the treatment of choice, given the multitude of factors influencing TT-TG measurement and distance, including trochlea dysplasia and femoral antetorsion [32, 33]. Although RL-TTO showed encouraging results, severe cartilage damage in the medial part of the patellofemoral joint during revision surgery was striking in this young patient cohort. Thus, further development of clinical outcomes needs to be awaited over a longer period of time. Overcorrection during TTO should be prevented by thorough planning of the amount of required medialization based on the preoperatively measured TT-TG and TT-PCL distances. In addition, medial overcorrection is unlikely with TTO self-centering techniques, while the reported complication rate remains low [3, 25, 34].

### Limitations

Only the short-term results of a small cohort of patients are presented in this study, and the findings must be confirmed to remain stable over a medium- to long-term follow-up period. However, no previous study that evaluated the outcome of RL-TTO is known to us. The majority of primary TTO surgeries were conducted outside of our institution, so it was not possible to determine exactly what the initial findings were that led to the decision to proceed with a medialization TTO. RL-TTO was not the only surgical procedure performed. The addition of different procedures (MPFL reconstruction, cartilage debridement/microfracturing, lateral retinacular lengthening) causes potential heterogeneity and possible bias to the results. Finally, the postoperative evaluation was performed using established PROMs and NAS assessment. However, the results were not verified by functional testing or imaging.

## Conclusion

RL-TTO significantly improved subjective knee function and AKP in patients suffering from medial patellar maltracking due to previous excessive tibial tubercle medialization osteotomy at short-term follow-up.
